# Genetic Basis of Antimicrobial Resistant Gram-Negative Bacteria Isolated From Bloodstream in Brazil

**DOI:** 10.3389/fmed.2021.635206

**Published:** 2021-03-15

**Authors:** Melise Chaves Silveira, Cláudio Marcos Rocha-de-Souza, Ivson Cassiano de Oliveira Santos, Leilane da Silva Pontes, Thamirys Rachel Tavares e Oliveira, Camila Bastos Tavares-Teixeira, Nataly de Almeida Cossatis, Natacha Ferreira Pereira, Orlando Carlos da Conceição-Neto, Bianca Santos da Costa, Daiana Cristina Silva Rodrigues, Rodolpho Mattos Albano, Fabrício Alves Barbosa da Silva, Elizabeth Andrade Marques, Robson Souza Leão, Ana Paula D'Alincourt Carvalho-Assef

**Affiliations:** ^1^Laboratório de Pesquisa em Infecção Hospitalar, Instituto Oswaldo Cruz - FIOCRUZ, Rio de Janeiro, Brazil; ^2^Departamento de Bioquímica, Instituto de Biologia Roberto de Alcântara Gome, Universidade do Estado do Rio de Janeiro – UERJ, Rio de Janeiro, Brazil; ^3^Programa de Computação Científica, Fundação Oswaldo Cruz - FIOCRUZ, Rio de Janeiro, Brazil; ^4^Departamento de Microbiologia, Imunologia e Parasitologia, Faculdade de Ciências Médicas, Universidade do Estado do Rio de Janeiro – UERJ, Rio de Janeiro, Brazil

**Keywords:** multidrug-resistance, bloodstream infections, gram-negative bacilli, whole-genome sequencing, Brazil, surveillance

## Abstract

Multidrug-resistant microorganisms are a well-known global problem, and gram-negative bacilli are top-ranking. When these pathogens are associated with bloodstream infections (BSI), outcomes become even worse. Here we applied whole-genome sequencing to access information about clonal distribution, resistance mechanism diversity and other molecular aspects of gram-negative bacilli (GNB) isolated from bloodstream infections in Brazil. It was possible to highlight international high-risk clones circulating in the Brazilian territory, such as CC258 for *Klebsiella pneumoniae*, ST79 for *Acinetobacter baumannii* and ST233 for *Pseudomonas aeruginosa*. Important associations can be made such as a negative correlation between CRISPR-Cas and *K. pneumoniae* CC258, while the genes *bla*_TEM_, *bla*_KPC_ and *bla*_CTX−M_ are highly associated with this clone. Specific relationships between *A. baumannii* clones and *bla*_OXA−51_ variants were also observed. All *P. aeruginosa* ST233 isolates showed the genes *bla*_VIM_ and *bla*_OXA486_. In addition, some trends could be identified, where a new *P. aeruginosa* MDR clone (ST3079), a novel *A. baumannii* clonal profile circulating in Brazil (ST848), and important resistance associations in the form of *bla*_VIM−2_ and *bla*_IMP−56_ being found together in one ST233 strain, stand out. Such findings may help to develop approaches to deal with BSI and even other nosocomial infections caused by these important GNB.

## Introduction

Multidrug-resistant (MDR) microorganisms are spread worldwide, and global efforts have been made on different fronts to reduce the incidence of MDR infections. Among them, strengthening antimicrobial resistance knowledge through surveillance and research is a major action (1).

Bloodstream infections (BSI) are usually associated with poor outcomes especially when adequate antimicrobial therapy and source control are delayed. Carbapenems have been used for the treatment of severe infections caused by gram-negative bacilli (GNB). However, carbapenem-resistant GNB, such as *Acinetobacter baumannii, Pseudomonas aeruginosa*, and *Enterobacterales*, are associated with decreased survival and have become a major challenge for therapeutics (2, 3). In this way, GNB bloodstream isolates are an important surveillance target for monitoring resistance and the World Health Organization (WHO) highlighted *A. baumannii, P. aeruginosa*, and *Klebsiella pneumoniae* as a critical priority to develop new antibiotics options (1).

Whole-genome sequencing (WGS) is a rapid molecular method for the search of molecular determinants of antimicrobial resistance (AMR). This tool has been proven to be fast and valuable to classify GNB in terms of resistance to the β-lactams and to guide antibiotic treatment decisions for BSI. WGS can detect not only important acquired genes but also chromosomal mutations, both of which contribute significantly to AMR (4). This approach improves our ability to analyze bacterial genomic content, and when these results are correlated with metadata and phylogenetic studies, we can understand with more precision the ways AMR bacteria multiply and spread (5).

Here, we conducted a surveillance about blood GNB isolates recovered from four of the five Brazilian regions using bioinformatic analysis of WGS data. The criteria and methods chosen are justified since as blood is a sterile site, these are more likely to represent true infections, and GNB is the group for which the highest resistance rates are currently found worldwide (2, 3). Phylogenetic analysis, clonal distribution, resistance mechanism diversity and other molecular aspects of nosocomial strains are described. Characteristics such as strain's national distribution, species diversity and the applied tools make this work the starting point for the construction of a new map of resistance in Brazil, contributing with global efforts to prevent the advance of AMR and assist in improving the therapeutic approach, especially for serious infections such as BSI.

## Materials and Methods

### Bacterial Strains

The Laboratório de Pesquisa em Infecção Hospitalar (LAPIH-FIOCRUZ) takes part in a National Bacterial Resistance Surveillance Network headed by The General Coordination of Public Health Laboratories (Brazilian Health Ministry). Our laboratory routinely receives clinical GNB isolates from hospitals located in different Brazilian states to confirm the mechanisms of drug resistance. The aim of this study was to identify the genetic basis AMR of selected bloodstream and catheter tip bacterial strains recovered from Brazilian states from January 2019 to September 2020.

All gram-negative isolates received by LAPIH in this period were submitted to carbapenemase genes detection by PCR (6) and colistin MICs by the broth microdilution method (http://www.eucast.org). The specie identification was confirmed by biochemical tests and for Complex *A. baumannii* isolates were performed the detection of *bla*_OXA−51_ gene by PCR.

Only isolates of *K. pneumoniae, A. baumannii*, and *P. aeruginosa*, the three serious cause of healthcare-associated infections and an emerging health threat worldwide, were selected to evaluate the antimicrobial resistance profile and to be submitted to WGS. The selection criterion per batch of isolates received over the period studied included representative carbapenemase-producing and/or polymyxin-resistant strains from different states.

### Antimicrobial Susceptibility

Antimicrobial susceptibility profiles were determined by an automated Vitek 2 system (bioMérieux-Vitek, Hazelwood, Mo).

### Genome Sequencing and Bioinformatic Analysis

WGS was performed using the Illumina Miseq (Illumina, San Diego, California, USA). A genomic library was constructed by transposon tagmentation with the Nextera XT DNA Sample Prep kit (Illumina, Inc., USA). Kraken2 was used to classify the reads taxonomically (7). The paired-end reads were *de novo* assembled using SPAdes v3.13.1 (8). Contigs shorter than 500 bp were discarded. CheckM was applied to assess the quality of genome sequencing and assembly, estimating their completeness, contamination, size and contigs parameters (9). The genome coverage was calculated using: coverage = (forward reads count ^*^ 2) ^*^ read length/total genome size. Species from *A. baumannii* were confirmed using Kmer-db (10) and reference strains (*A. baumannii* KL810966.1, *A. nosocomialis* GCF_000368085.1, *A. pittii* CP002177, and *A. calcoaceticus* NZ_LS999521).

Draft genomes were then annotated using Prokka (11). The prediction of the resistome and plasmid incompatibility (Inc) group were made using the ABRicate (T. Seemann, https://github.com/tseemann/abricate) against ResFinder (12), and PlasmidFinder (13), respectively. Only the results above 90% of coverage and identity using ABRicate were considered. Besides that, BLAST (blastx) alignment results were analyzed to identify mutations related to resistance (14). The proteins analyzed were PhoP and PhoQ (polymyxin resistance), besides RamR and AdrR (tigecycline resistance) for *K. pneumoniae*, using MGH78578 as reference; ColR, ColS, and PhoQ (polymyxin resistance), besides OprD (carbapenem resistance), AmpC (cephalosporin resistance) and MexT (different classes) for *P. aeruginosa* using PA01 as reference (AAG07497.1, the functional variant CAA07694 being used for MexT); and LpxA, LpxC, and LpxD (polymyxin resistance), besides AdeN and AdeR (tigecycline resistance) for *A. baumannii* using ATCC 19606 as reference. Furthermore, proteins PmrA and PmrB (polymyxin resistance), and GyrA, GyrB, and ParC (fluoroquinolones resistance) were analyzed for all these three species. Deleterious mutations were checked using PROVEAN web server (15). We only considered mutations classified as deleterious in Results and Discussion section. Presence of a CRISPR-Cas system was assessed by the CRISPRCasFinder (16). MLST was performed with the MLST software (T. Seemann, https://github.com/tseemann/mlst) and the PubMLST database (17).

Phylogenetic analysis were performed using kSNP3.0 software, without core genome option (18). The program Kchooser, which is part of the kSNP package, was used to identify the optimal kmer length. iTOL was applied for visualizing and annotating the phylogenetic trees (19).

### Genome Accession Number

All draft genomes are available at GenBank BioProject accession PRJNA677881.

## Results

In the period of January 2019 to September 2020, 577 blood GNB isolates were received by LAPIH to characterize the resistance mechanisms (Supplementary Table 1). The isolates belonged to different species, the most frequent were *K. pneumoniae* (*n* = 200), *A. baumannii* (*n* = 192), and *P. aeruginosa* (*n* = 119). PCR analysis showed that the most prevalent carbapenemase gene among Enterobacterales isolates was *bla*_KPC_ (69,6%, *n* = 176) and among *Acinetobacter* species was *bla*_OXA−23like_ (89,6%, *n* = 172). Finally, *Pseudomonas* isolates were found to carry *bla*_VIM_ (*n* = 20), *bla*_KPC_ (*n* = 17), *bla*_IMP_ (*n* = 8), and *bla*_SPM_ (*n* = 4). However, 63.0% (*n* = 75) of *P. aeruginosa* isolates did not carry any of the investigated carbapenemases. Excluding species intrinsically resistant, polymyxin resistance was detected in 170 isolates (30.7%), belonging mainly to *K. pneumoniae* (*n* = 123/200, 61.5%). Furthermore, the plasmid-mediated *mcr-1* gene was found in 2 *Escherichia coli*.

Based on the results obtained, WGS was performed on 84 selected isolates and included the following species: *K. pneumoniae* (*n* = 46/200, 23.5%, being one *K. quasipneumoniae*), *A. baumannii* (*n* = 23/192, 12%), and *P. aeruginosa* (*n* = 15/119, 12.6%). These isolates were representative of carrying carbapenemase genes and/or polymyxin resistance recovered from the 7 Brazilian states included in the study belonging to 4 geographic regions: Northeast (51.2%), Southeast (35.7%), Midwest (6%), and North (7.1%) (Supplementary Table 2).

**Figure 1:**
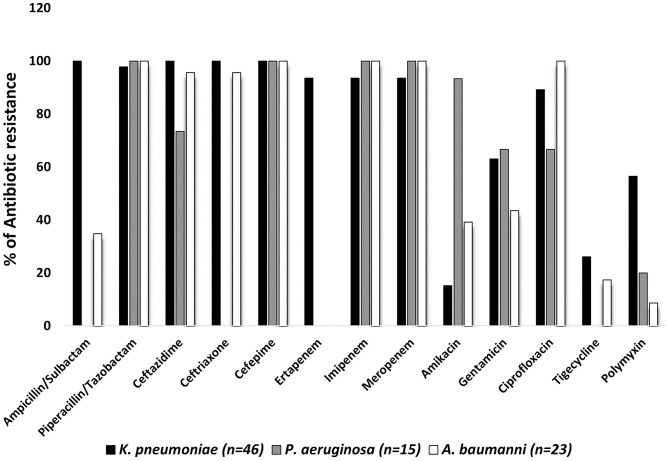
Antibiotics resistance patterns in percentages from selected bloodstream GNB isolated from January 2019 to September 2020. Antimicrobial susceptibility tests were interpreted according to the European/Brazilian Committee on Antimicrobial Susceptibility Testing guidelines. The minimum inhibitory concentration for colistin was obtained using the broth microdilution test, according to the European/Brazilian Committee on Antimicrobial Susceptibility Testing guidelines.

Antimicrobial resistance levels for the sequenced organisms are shown in Figure 1. All strains from the three species analyzed displayed resistance to cefepime. Most significantly, the vast majority of the *K. pneumoniae, A. baumannii*, and *P. aeruginosa* isolates showed resistance to piperacillin-tazobactam, imipenem, and meropenem (±98.33%). Ampicillin-sulbactam, ceftazidime, and ceftriaxone were not active against all *K. pneumoniae* strains and the resistance rates for ertapenem, ciprofloxacin, gentamicin, tigecycline, and amikacin were 93.43, 89.13, 63.04, 26.08, and 15.22%, respectively. The *P. aeruginosa* isolates showed high resistance to amikacin, ceftazidime, gentamicin, and ciprofloxacin (93.33, 73.33, 66.66, and 66.66%, respectively). Furthermore, the antibiotics resistance pattern for *A. baumannii* isolates was: ciprofloxacin (100%), ceftriaxone (95.65%), ceftazidime (95.65%), gentamicin (43.48%), amikacin (39.13%), ampicillin-sulbactam (34.48%), and tigecycline (17.39%). Finally, 27/46 (56.46%) *K. pneumoniae*, 3/15 (20%) *P. aeruginosa* and 2/23 (8.69%) *A. baumannii* isolates showed polymyxin resistance (MICs >2mg/L) by broth microdilution.

**Figure 2:**
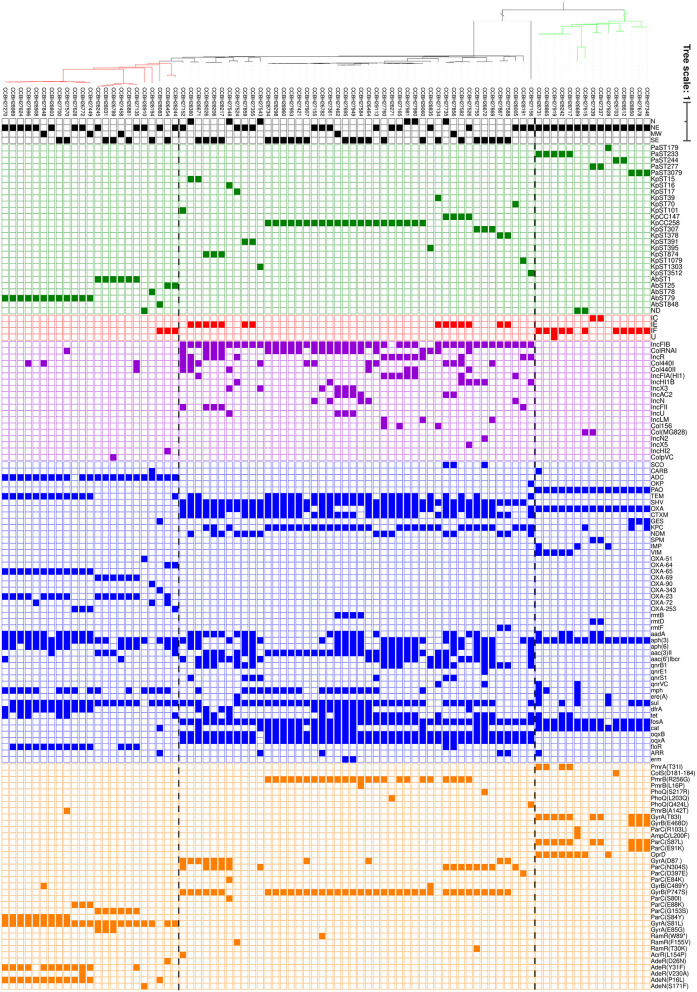
Whole-genome SNP-based parsimony tree including isolates of *K. pneumoniae* (46 isolates, black clade), *A. baumannii* (23 isolates, red clade), and *P. aeruginosa* (15 isolates, green clade) generated by kSNP3.0. The branch lengths are expressed in terms of changes per number of SNPs. The tree was visualized using iTol (19). The panel shows the presence (filled squares) and absence (empty squares) of the characteristics analyzed. Each color corresponds to a characteristic: black (Brazilian regions), green (Sequence Types), red (Cas type), purple (Inc groups), blue (resistance acquired genes), and orange (deleterious mutations in proteins related to antimicrobial resistance). N, north; NE, northeast; MW, Midwest; SE, southeast; ND, not determined.

The bioinformatic analysis results are summarized in Figure 2. The acquired genes found are related to resistance to 11 different classes, including beta-lactams, aminoglycosides, sulfonamides, fluoroquinolone, macrolide, trimethoprim, tetracycline, fosfomycin, chloramphenicol, rifamycin, and macrolide/streptogramin/lincosamide (MLS phenotype). Most of the sequenced strains were positive for gene families *sul* (76.2%, sulfonamides resistance), *aph(3)* (73.8%, aminoglycosides resistance), and *fosA* (71.4%, fosfomycin resistance). Fifteen families of β-lactamase genes were found (*bla*_KPC_, *bla*_NDM_, *bla*_SPM_, *bla*_IMP_, *bla*_VIM_, *bla*_CTX−M_, *bla*_GES_, *bla*_TEM_, *bla*_SHV_, *bla*_SCO_, *bla*_CARB_, *bla*_ADC_, *bla*_OKP_, *bla*_PAO_, and *bla*_OXA_).

The most prevalent resistance genes among *K. pneumoniae and K. quasipneumoniae* isolates were *fosA* (100%), *bla*_SHV_ (97.8%), and *oqxAB* (97.8%, fluoroquinolones resistance). Plasmids from the incompatibility group IncFIB were found in 93.5% of all isolates. The strains belonged to 20 STs. The CC258 (ST11, *n* = 16, ST258, *n* = 4, and ST437, *n* = 1) was the most prevalent clonal complex (45.7%) found in four Brazilian regions, followed by CC147 (ST147, *n* = 1, ST392, *n* = 2, and ST273, *n* = 1) (8.7%). All CC258 strains analyzed showed absence of CRISPR-Cas and 85.7% were found to carry the genes *bla*_KPC_, *bla*_CTX−M_, and *bla*_TEM_. The *rmtB* gene (aminoglycosides resistance) was detected solely in ST258, in 4 strains. Of these, three are the only amikacin resistant strains from CC258. IncA/C2 was found only in ST258 strains. The CC258 showed a high Inc groups diversity, being IncFIB the most prevalent (90.5%), followed by ColRNAI (66.7%) and IncR (28.6%). In addition, deleterious mutations were identified in ParC (S80I) for all CC258 strains and in PmrB (R256G) for 90.5% of them. ParC could interfere in fluoroquinolones resistance, and indeed all CC258 strains were ciprofloxacin resistant. On the other hand, 5 *K. pneumoniae* strains from CC258 were polymyxin-susceptible despite the presence of mutation in PmrB (R256G). The CC258 strains with PhoQ (L203Q) or PmrB (L16P) deleterious mutations were resistant to polymyxin. All CC147 strains showed the adaptative immune system CRISPR-Cas type IE and were found to carry the resistance genes *bla*_OXA−1_, *bla*_SHV_, *oqxAB*, and *sul1*. IncFIB and deleterious mutations in PmrB (R256G), ParC (S80I), and ParC (N304S) were also found in all strains. Despite PmrB mutation, 75% of CC147 strains were polymyxin susceptible. In contrast, all of them were ciprofloxacin resistant and it could be related to ParC mutations. The ST3512 *K. quasipneumoniae* strain (CCBH27156), presented some unique characteristics, such as the simultaneous presence of the *bla*_KPC_ and *bla*_NDM_, absence of the *bla*_SHV_, and mutation in PhoQ (Q424L), however, it was not associated with polymyxin resistance. Among all strains with PmrB R256G mutation, 33.3% were susceptible to polymyxin. The deleterious (T30K) and non-sense (W89^*^) mutations on RamR were only present in tigecycline resistant strains. Another 10 strains proved to be tigecycline resistant without any characteristic mutation identified. The *tet(x)* gene, related to tigecycline resistance, was not found.

The 23 *A. baumannii* strains analyzed belonged to 6 STs and were recovered from three Brazilian regions. ST79 was the most prevalent ST found (52.2%), followed by ST1 (26.1%) and ST25 (8.7%). The most common characteristics were the deleterious mutation S81L in GyrA (95.7%), and the resistance genes *bla*_ADC_ (95.7%), *aph(3)* (91.3%), and *sul* (91.3%). The resistance genes *bla*_OXA−65_ (*bla*_OXA−51_ allelic variant), *bla*_TEM−1A_, and *dfrA* (trimethoprim resistance) were only detected in all ST79 strains. The *bla*_OXA−23_ was the most prevalent carbapenemase gene among ST79 strains (66.7%). The carbapenemase gene *bla*_OXA−253_ (*bla*_OXA−143_ allelic variant), was also detected in three strains from this ST. The resistance gene *bla*_OXA−69_ (*bla*_OXA−51_ allelic variant), and the deleterious mutation G153S in ParC were only found in all ST1 strains. The carbapemase gene *bla*_OXA−23_ was also found in all ST1 strains. A few *A. baumannii* strains presented the Inc groups investigated (26.1%). A IncHI2 plasmid and the carbapenemase genes *bla*_OXA−23_ and *bla*_OXA−72_ (*bla*_OXA−24_ allelic variant), were found in one strain belonging to ST25. The CRISPR-Cas system type IF was detected in only three strains, assigned to ST25 and ST848. Interestingly, the only ST848 strain presented *bla*_GES−11_ and *arr* genes (rifamycin resistance) and the absence of S81L mutation in GyrA, differing from the other *A. baumannii* isolates. The only strain with a PmrB deleterious mutation showed polymyxin susceptibility (A142T in CCBH27570). We did not identify deleterious mutation associated to the two polymyxin resistant strains. The mutations AdeR(D26N), AdeR(V230A), and AdeN(S171F) were found in tigecycline-susceptible strains. Two mutations were majority related to ST79 (AdeR, Y31F and AdeN, P16L), present in both susceptible and resistant strains. The *tet(x)* gene was not found.

The 15 *P. aeruginosa* strains analyzed belonged to 5 STs and were from 1 Brazilian region. ST233 (33.3%) was the most prevalent ST found, followed by ST3079 (20%). All strains showed the resistance genes *bla*_PAO_, *aph*(3), and *catB7* (chloramphenicol resistance). The CRISPR-Cas system was detected in 86.6% of the strains, being type I-F the most frequent. Deleterious mutations T83I and S87L in GyrA and ParC, respectively, were the most frequently found (66.7%), and were distributed in different clones. These two mutations were present in all ciprofloxacin resistant strains and absent in strains susceptible to this antibiotic. All 5 ST233 strains showed the genes *bla*_VIM_ and *bla*_OXA486_. The PmrA deleterious mutation T31I was found in four strains belonging to this ST, and two of them are resistant to polymyxin according to MIC. Furthermore, one ST233 isolate carried the carbapenemase genes *bla*_VIM−2_ and *bla*_IMP−56_ together. The three ST3079 strains were the only ones to show the gene *bla*_GES−1_ and the deleterious mutations in Parc (E91K) and GyrB (E468D). The genes *bla*_SPM_, *bla*_OXA−56_, and *rmtD* were found only in all ST277 isolates. A strain from ST244 with ColS amino acids deletion (D181–184) was polymyxin susceptible. The plasmid incompatibility group Col(MG828) was found in two *P. aeruginosa* isolates from different STs.

## Discussion

In the present study we analyzed gram-negative pathogens causing BSI and *K. pneumoniae* was the major species among MDR strains. A national study about nosocomial BSI in Brazil reveled that monomicrobial episodes of BSI were mostly caused by gram-negative bacteria, highlighting *K. pneumoniae* (20). Other Brazilian data about BSI, from a single hospital, showed that *K. pneumoniae* infections had a positive association with the MDR phenotype (21). Both studies corroborate the high prevalence of this specie among the isolates selected for the present work.

Antibiotic resistance within the group of gram-negative bacteria related to BSI is worrisome in Brazil, and the pathogens analyzed here are among the most outstanding (20, 21). According to our results, amikacin was the most effective antimicrobial against *K. pneumoniae*, and, *A. baumannii* and *P. aeruginosa* showed a great sensibility to polymyxin.

A predominance of worldwide distributed clones among *K. pneumoniae* (CC258), *A. baumannii* (ST79) and *P. aeruginosa* (ST233) strains was observed. Isolates from international high-risk CC258 have been found in all Brazilian states and are highly associated with the dissemination of carbapenemase KPC (6, 22). The ST1 is designed as the second major *A. baumannii* clone and is globally associated to OXA-23 (23), however in South America this carbapenemase has been most commonly related with ST79, the third major international distribution clone, as noted here (23, 24). The most prevalent ST in *P. aeruginosa*, ST233, is a worldwide disseminated MDR clone particularly linked to VIM-2, as was corroborated by our results (25).

The prevalence of gram-negative BSI has practical importance, especially when dealing with the issue of treatment, creating a vicious cycle of antimicrobial heavy use and resistance development (20). A great variety of resistance genes was found, highlighting *sul* and *fosA*. High prevalence rates of *sul* variants have been observed mainly in gram-negative bacteria isolated all over the world (26). Meanwhile, *fosA* is commonly found in *K. pneumoniae* and *P. aeruginosa* contributing to intrinsic fosfomycin resistance, but is largely absent in *A. baumannii* (27), as confirmed here. The resistance rates to carbapenems equal or higher than 93,48% can be associated to specific carbapenemases genes found more often. The carbapenemase *bla*_KPC−2_ was very prevalent in *K. pneumoniae*, contrasting with *bla*_NDM−1_. This difference is expected once *bla*_KPC−2_ circulates in Brazil since 2009 (28), and *bla*_NDM−1_ was detected only in 2013 in this country (29). Furthermore, most of the analyzed strains are CC258 *K. pneumoniae*, which is spread in Brazil mainly carrying *bla*_KPC_ (22). The most prevalent carbapenemase in *A. baumannii* strains, OXA-23, was detected here in already associated clones as ST1, ST25 and ST79 (23, 30). Although this gene was not detected in some ST79 strains from this surveillance, all of them have OXA-65 carbapenemase, as previously reported in a Brazilian surveillance (31). The single strain from *A. baumannii* ST848 calls to attention since the occurrence of *bla*_GES−11_, which mediates resistance to β-lactam and reduced susceptibility to carbapenem, has been reported in other continents and clones but neither in America nor in ST848 (32). Furthermore, this clone is not clearly associated with *A. baumannii* isolates from Brazil. The carbapenemases genes found in *P. aeruginosa* are highly associated with a specific clone. The association of *bla*_VIM−2_ to ST233 (25) and *bla*_SPM−1_ to ST277 (33) have been reported. On the other hand, the association of ESBL *bla*_GES−1_ to ST3079 has not yet been described. Carbapenemase KPC-2 was also present in 2 of the 3 strains analyzed from this clone. It is important that the dispersion of *P. aeruginosa* ST3079 be monitored and the prevalence of these important genes confirmed. The coexistence of genes *bla*_VIM_ and *bla*_IMP_ in *P. aeruginosa* found here for a single strain was described before in India, associated with a MIC for meropenem higher than 32 μg/ml (34), so this association also demands attention.

Since resistance to carbapenems is usually associated with multidrug resistance, polymyxins became the last alternative (20). Here we detected different deleterious mutations in PhoQ for *K. pneumoniae*, PmrA for *P. aeruginosa* and PmrB for *K. pneumoniae* and *A. baumannii*. It is difficult to extrapolate whether some substitutions identified in proteins, mainly in *P. aeruginosa* and *A. baumannii*, leads to polymyxin resistance, and the levels of gene expression may vary and consequently influence the level of resistance (35). The deleterious mutation R256G in PmrB which seems common in *K. pneumoniae* CC258 and CC147, has been found in polymyxin-susceptible isolates (36), and the same was observed here. Other studies will be necessary to verify if PhoQ (L203Q) and PmrB (L16P) deleterious mutations are indeed associated to polymyxin resistant in *K. pneumoniae*. As far as we know, the PmrA deleterious mutation shown in *P. aeruginosa* (T31I) has not yet been linked to polymyxin resistance, but among the 4 strains that showed this mutation, 2 were resistant according to MIC. The PmrB A142T mutation in *A. baumannii* is present in a susceptible strain, and has not yet been linked to polymyxin resistance.

Although ParC deleterious mutations occurred in different *K. pneumoniae* clones and may cause ciprofloxacin resistance, we could not ignore the high frequency of *oqxAB* genes, which encode a efflux pump that confer resistance to multiple agents including fluoroquinolones. Some mutations observed for *P. aeruginosa* are relevant examples of gain-of-function and are associated with resistance to fluoroquinolones [GyrA (T83I), GyrB (E468D) and ParC (S87L)] (37). Indeed, we showed that the GyrA and ParC deleterious mutations were strict associated to ciprofloxacin resistance. For *A. baumannii*, the simultaneous presence of mutations in GyrA (S81L) and in ParC (S84L) have been associated to resistance to ciprofloxacin and nalidixic acid (38). All *A. baumannii* strains analyzed here are ciprofloxacin resistant and most of them have the GyrA S81L mutation (95.8%). For ST1, the deleterious mutations ParC (S84L e G153S) could be noted simultaneously to GyrA(S81L), and for ST79 the concomitant mutation was ParC(S84Y). These associations could explain ciprofloxacin resistance for the major *A. baumannii* STs in this study.

Plasmid-mediated transfer of resistance has led to widespread dissemination, outbreaks, and untreatable infections. Molecular identification of plasmid and strain genotypes can distinguish whether the spread of AMR genes is driven by epidemic plasmids to different hosts or by clonal spread of bacterial organisms harboring these plasmids with AMR genes (39). Here, the diversity and abundance of Inc groups found in *K. pneumoniae* strains contrast with *P. aeruginosa* and *A. baumannii* results. The majority of *K. pneumoniae* strains analyzed have the IncFIB plasmid, a group known for their MDR characteristic (39, 40). In Brazil, the IncFIB plasmid has already been described as carrying *bla*_KPC−2_ and *bla*_NDM−1_ in *K. pneumoniae* (40, 41). A recent manuscript reported that plasmids with IncR, ColRNAI and IncF co-exists in KPC-2-producing *K. pneumoniae* strains from ST11 (42). Interestingly, we detected IncR in most ST11 isolates, but not in other CC258 clones. IncN was detected mainly in CC258, which is expected as in Brazil the spread of *bla*_KPC−2_ has already been related to dispersion of Tn4401 'b', carried by IncN plasmids mainly disseminated by this clonal complex (22). IncA/C2 was only detected in ST258 strains, the same strains which carry *rmtB* and *tet(G)* genes. This profile is very similar to plasmid pMTY16641, identified in the first KPC-producing *K. pneumoniae* ST258 isolated from a Japanese patient without a history of foreign travel (43). The incompatibility group IncHI2 was found in a single *A. baumannii* strain, the only one positive both for *bla*_OXA−23_ and *bla*_OXA−72_, and as far as we know, this group has not yet been associated with such genes.

The only species for which almost all strains present CRISPR-Cas systems was *P. aeruginosa*. Types I-F and I-C identified here were already described in *P. aeruginosa* strains. Type I-F is the most widely distributed system among *P. aeruginosa*, while most type I-C-positive strains seem restricted to MDR clones, like ST277 (44), and a similar scenario was observed here. Only CRIPR-Cas type I-E is already described in the literature for *K. pneumoniae*, as was seen in this surveillance (45). It was also confirmed that this type is extremely rare in CC258 (45). However, other linages positive for *bla*_KPC_, like CC147 and ST874, presented half or more positive strains for type I-E CRISPR-Cas system, so the negative association between CRISPR-Cas system and *bla*_KPC_ seems to be specific to CC258. In *A. baumannii*, only the CRISPR type I–F system has been found so far (46), and we detected it in 12.5% of the strains. One of these was also positive for IncHI2 contradicting the recently reported negative association between *A. baumannii* strains with CRISPR system and plasmid detection (46).

WGS is a powerful tool to provide reliable data for monitoring clonal dispersion, antimicrobial resistance and other related characteristics. In the presented study, it was possible to highlight high-risk clones circulating in Brazilian territory. Some important associations could be made between these clones, important resistance genes and specific deleterious mutations in genes also related to antibiotic resistance. In addition, some trends could be identified such as new MDR clones, and novel important resistance genes and clonal profiles that are circulating. Such findings may help to develop approaches to deal with BSI and even other nosocomial infections caused by these important GNB, mainly in Brazil but also in other countries, since most clones are globally dispersed.

## Data Availability Statement

The datasets presented in this study can be found in online repositories. The names of the repository/repositories and accession number(s) can be found below: https://www.ncbi.nlm.nih.gov/bioproject/?term=PRJNA677881.

## Author Contributions

AC-A conceived the research. IO, LP, TO, CT-T, NC, NP, OC-N, BC, and DR were responsible for the execution of phenotypic and molecular tests to identify and detect resistance of Gram-negative bacteria. AC-A and CR participated in the selection of sequenced strains. MS, CR, and RL participated in method design, whole genome sequencing, and data handling. MS performed the data analysis. MS and CR made the figures. AC-A, MS, CR, RA, FS, EM, and RL wrote parts and edited the complete manuscript. All authors have read and approved the manuscript.

## Conflict of Interest

The authors declare that the research was conducted in the absence of any commercial or financial relationships that could be construed as a potential conflict of interest.

## Supplementary Material

Supplementary Table 1 PCR and MIC results about the 577 blood GNB isolates received between January 2019 to September 2020.

Supplementary Table 2 General information about 84 blood GNB isolates submitted to whole genome sequencing.
